# Relationship between Malnutrition and the Number of Permanent Teeth in Filipino 10- to 13-Year-Olds

**DOI:** 10.1155/2013/205950

**Published:** 2013-08-29

**Authors:** Roswitha Heinrich-Weltzien, Carsten Zorn, Bella Monse, Katrin Kromeyer-Hauschild

**Affiliations:** ^1^Department of Preventive and Paediatric Dentistry, Jena University Hospital, WHO Collaborating Centre for Prevention of Oral Diseases, Bachstraße 18, 07743 Jena, Germany; ^2^Deutsche Gesellschaft für Internationale Zusammenarbeit (GIZ) GmbH, GIZ Office Manila, PDCP Bank Centre, V.A. Rufino Corner L.P. Leviste Street, Makati, 1007 Metro Manila, Philippines; ^3^Institute of Human Genetics, Jena University Hospital, Kollegiengasse 10, 07743 Jena, Germany

## Abstract

In the present study, we determined whether there is a delay in the eruption of permanent teeth (PT) among Filipino adolescents with stunting or thinness. Height, weight, and number of PT were recorded in 1554 Filipino 10- to 13-year-olds (711 boys; 843 girls). *z*-scores for height (HAZ) and body mass index (BMI) were calculated according to the WHO growth reference, and their correlations to the number of PT were assessed. 54.9% of the children have at least one form of malnutrition. Significantly, more boys (22.9%) than girls (16.5%) were thin, while no sex difference in stunting was noted (boys 48.5%; girls 44.0%). The number of PT was significantly correlated to HAZ and BMI-*z*-score. Stunted and thin students had significantly fewer PT than their nonaffected peers. These differences tended to be the result of delay in tooth eruption in thin and stunted adolescents. In 13-year-old girls, all PT were erupted regardless of their nutritional status indicating a catch-up. Thin and stunted boys had one tooth less than normal boys at this age. Impaired physical growth and dental development seem to have common risk factors. Therefore, regular monitoring of growth and dental development might be helpful for targeting support programmes in developing countries.

## 1. Introduction

In developing countries, malnutrition in the form of undernutrition is a major health concern and the most common cause of growth failure in children. According to the World Health Organization (WHO), malnutrition “signifies an imbalance between the supply of protein and energy and the body's demand for them to ensure optimal growth and function” [1, 2]. In this context, malnutrition is synonymous with protein-energy malnutrition (PEM). The balance can be either negative leading to undernutrition or positive leading to overweight and obesity. However, this article refers to those who are malnourished due to undernutrition.

Malnutrition in children is often called the “syndrome of developmental impairment” since in addition to growth failure other impairments, such as delayed motor, cognitive, and behavioural development, and increased morbidity and mortality, may occur. 

Wasting (low weight-for-height—indicating acute episodes of malnutrition), or much more common, stunting (low length- or height-for-age—resulting from chronic malnutrition) are tools for evaluation of malnutrition. For community studies, Waterlow [2] defined categories of PEM according to the grading of severity. This quantitative classification differentiates between mild (87.5–95% stunting or 80–90% wasting), moderate (80–87.5% stunting or 70–80% wasting), and severe (<80% stunting or <70% wasting) categories. For severely undernourished children, Waterlow suggested an additional qualitative classification to distinguish marasmus, kwashiorkor, and intermediate forms [2]. In the WHO Global Database on Child Growth and Malnutrition, a cutoff point of <−2 SD is used to classify low weight-for-age, low height-for-age, and low weight-for-height as moderate and severe undernutrition, and <−3 SD to define severe undernutrition [1].

Nutritional deficiencies (insufficient intake of energy, proteins, and micronutrients) may influence the skeletal as well as the dental development because the role of nutrition for oral tissues may not be different from that for other tissues and organ systems. The relationship between nutritional status and oral health has become a subject of increased research over the past two decades [3–5]. It has been shown that tooth was positively correlated with different measures of somatic growth and maturity [6]. Due to methodical differences (e.g., definition of nutritional status, nutritional status range, primary or permanent dentition, sex, age, and ethnic group), correlation coefficients between dental development and nutritional status vary between studies. Whereas correlations of low or intermediate magnitude were found in the Fels Longitudinal Study [6, 7], other studies [8–10] reported a close correlation.

Several cross-sectional studies have shown that various nutritional deficiencies, including calcium [11], vitamin D [12], vitamin B_12_ [13], and ascorbic acid [14], may be related to different forms of periodontal disease or oral manifestations and symptoms. The first study investigating the relationship between early childhood PEM and periodontal status among Haitian 12- to 19-year-olds revealed that more than half of the adolescents exhibited periodontal pockets of 4-5 mm or greater in at least one sextant [15]. These findings indicate that periodontitis in the permanent dentition may be predisposed by early childhood PEM. Results from studies focused on the effects of malnutrition on timing of tooth formation are conflicting. While some studies did not find a significant delay in tooth growth in undernourished children [16, 17], others suggested a significant effect of malnutrition on dental development [18, 19]. Furthermore, many studies with various designs provided a significant body of evidence that PEM and enamel hypoplasia in the primary dentition are associated, while the association in the permanent dentition is less substantiated (see review from Psoter et al. [20]). To date, there is little information relating malnutrition to the eruption of permanent teeth (PT). Whereas Toverud [21] reported a delayed eruption of PT in malnourished children (however not attributing this finding to nutritional stress), Alvarez [22] observed an accelerated eruption of the permanent incisors and first molars. Psoter et al. [20] discussed the limitations of these two longitudinal studies, and concluded that no firm association between early childhood PEM and the age of eruption of PT exists. However, a more recent retrospective cohort study presented evidence of an association between childhood severe chronic malnutrition and delayed exfoliation of primary teeth as well as delayed eruption of PT [23]. Thus, disturbance in tooth eruption with altered number of teeth may indicate an altered physical development and vice versa; an altered physical development may be related to a disturbance in tooth eruption. Both parameters are valuable indicators of public health. The purpose of this study was to assess the relation between the number of erupted PT and the nutritional status in 10- to 13-year-old students from rural areas in the Philippines where malnutrition is still a major health concern. It should be examined whether there is a delay in the eruption of PT among Filipino adolescents with stunting or thinness.

## 2. Materials and Methods

### 2.1. Study Area and Sample

The data were collected during the final evaluation of a dental preventive programme for Filipino elementary school children initiated in 1998 by the German nongovernmental organization (NGO) “German doctors” with a running time of 5 years. The Philippine Department of Education, Culture and Sports selected 19 elementary schools located in rural areas of the province Misamis Oriental, Mindanao, in southern Philippines, to participate in this programme. In 2000, further 13 schools were included. Because of the poor oral health status of Filipino children, the programme focused on primary (daily toothbrushing with fluoride toothpaste, application of a fluoride varnish twice a year, and dietary counseling) as well as tertiary (restorative treatment of permanent teeth, extraction of nonrestorable primary, and PT) preventive procedures. Only first graders of the selected schools participated in the programme. The parents of the children were informed about the programme and gave written informed consent. This survey was carried out from July to August 2003. Within the total sample of 1764 10- to 16-year-olds, this report is based on 1554 students aged 10 to 13 years at the time of the field examination in the summer of 2003 and had complete data. The criterion of only including 10- to 13-year-olds, that is, less than 14-year-olds, in this analysis was employed to capture the established eruption period for the permanent canines, first and second premolars, and second molars. Children who received primary tooth extractions were excluded from the data analysis because it could have altered the eruption timing of the successor permanent tooth. When PT had been extracted in the context of dental treatment, they were added to the overall number of PT for the purpose of this study.

### 2.2. Ethical Approval

The dental preventive programme as well as the evaluation examination received an approval from the Ethics Committee of the Xavier University of Cagayan de Oro, Philippines. Written parental/legal guardian consent was obtained prior to the participation in the dental preventive programme and the examination. 

### 2.3. Oral Examination

The examination was performed outside in the schoolyard with sunlight as a direct light source. After brushing their teeth, the students were placed in a supine position on a long classroom bench, with their heads on a pillow on the lap of the examiner, who sat behind them. Tooth eruption was defined as having occurred if any tooth surface had pierced the alveolar mucosa. 

To ensure consistent clinical judgment, all 9 dental examiners and recorders underwent 2 days of training by a WHO consultant epidemiologist, who was seen as the gold standard. Reexamination of every 20th subject throughout the study was performed. PT counting was part of the caries assessment training.

### 2.4. Anthropometric Measures

Height of the children erected and without shoes was measured with a portable stadiometer (Seca 216 Height Rod; Seca GmbH & Co. KG, Hamburg, Germany) to the nearest 0.5 cm. Weight was measured with children wearing minimal light clothing using a portable electronic digital scale (Soehnle Gala XL; leifheit AG, Nassau, Germany) to the nearest 0.5 kg. No adjustments were made for clothing. The measuring equipment was recalibrated daily on arrival in school, as a routine part of the field operations. All measurements were carried out by well-trained nurses following standardized guidelines [24]. The mean interobserver difference was 2.5 mm (±1.9 mm) in height. Since weight was measured with near-perfect precision on a digital scale, it was not included in the training sessions. 

Body mass index (BMI) was computed as weight/height^2^ (kg/m^2^). Height-for-age and BMI-for-age were converted to *z*-scores based on the 2007 WHO growth reference for school-aged children and adolescents [25]. Thinness was defined as a BMI-for-age less than 2 SDs from WHO reference medianvalue; similarly, children whose height-for-age *z*-score (HAZ) was less than −2 were stunted. An SD value of −2 corresponds to the 2.3th percentile, that is, 2.3% of the reference population is measured lower than SD value of −2.

### 2.5. Statistical Analysis

Data were analyzed using SPSS, version 18.0. Anthropometric and dental characteristics were presented as mean (and standard deviations) or as median values (and interquartile ranges dependent on their distribution). The Kolmogorov-Smirnov test revealed that in boys and girls only the distribution of height did not show statistically significant departure from normality at any age. Prevalences were given as percentage (and confidence intervals). *t*-test (height), Mann-Whitney *U* test, and *χ* ^2^-test were used to compare these variables between sexes. Mann-Whitney *U* test was used to compare the number of PT expressed as median values (P25; P75) in the nutritional status categories. The relations between the number PT and BMI-*z*-scores as well as height-*z*-scores were assessed by Spearman's rank-order correlations. The level of significance was set at *P* ≤ 0.05.

## 3. Results

The study included a sample of 1554 students aged 10 to 13 years. Study characteristics stratified by sex are shown in Table 1. The interexaminer kappa values for counting the number of PT and caries scoring were in the same range of *κ* 0.78–0.92, and the intraexaminer reproducibility ranged between *κ* 0.87 and 0.97, which can be classified as good. 

Height, weight, and BMI were significantly higher in girls than in boys. Girls had also significantly more PT than boys, which suggests an earlier permanent dentition in females than in males. Mean HAZ was −1.9 (±1.1 boys; ±1.3 girls) in both sexes, indicating high rates of stunting. No significant difference in the prevalence of stunting was noted between boys and girls, whereby little more boys (48.5%) than girls (44.0%) tended to be stunted. Approximately, one fifth of the students were thin (BMIZ < −2). The prevalence of thinness was significantly higher in boys (22.9%) than in girls (16.5%). Boys had also a significantly lower mean BMI-*z*-score (−1.2 ± 1.3) than girls (−1.0 ± 1.3). Stunting among thin children was high (50.9% in boys and 59.0% in girls), but only one fifth of the stunted students (24.1% in boys and 22.1% in girls) were thin.

**Figure 1:**
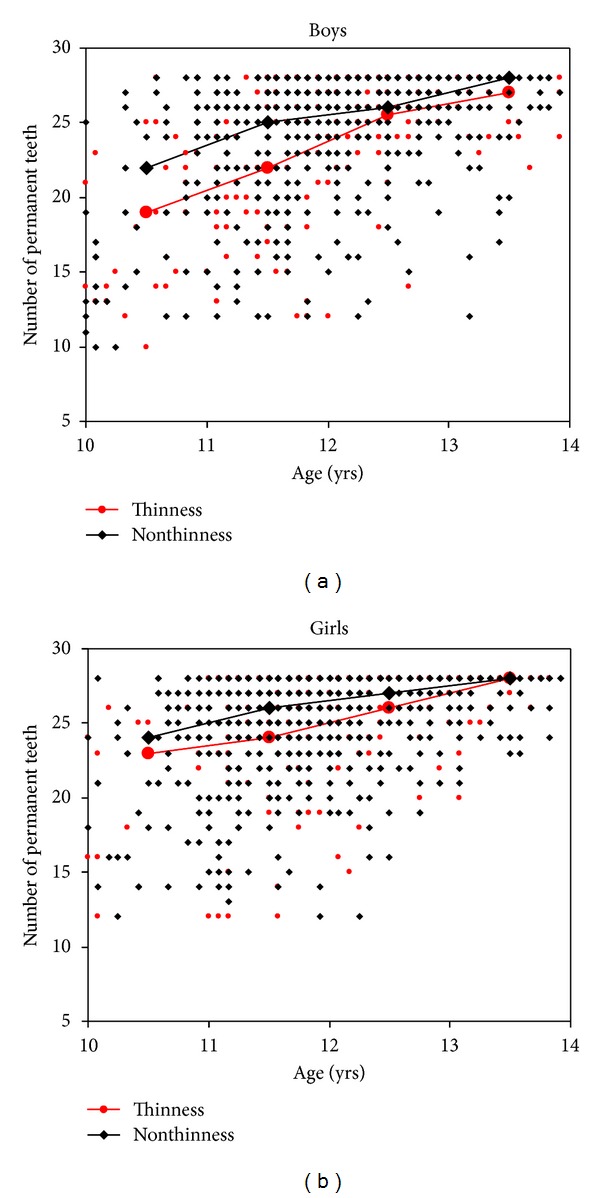
Relationship between number of permanent teeth and age in thin and nonthin (a) boys, and (b) girls with interpolation lines between median values in age groups (the midpoint of the interval).

**Figure 2:**
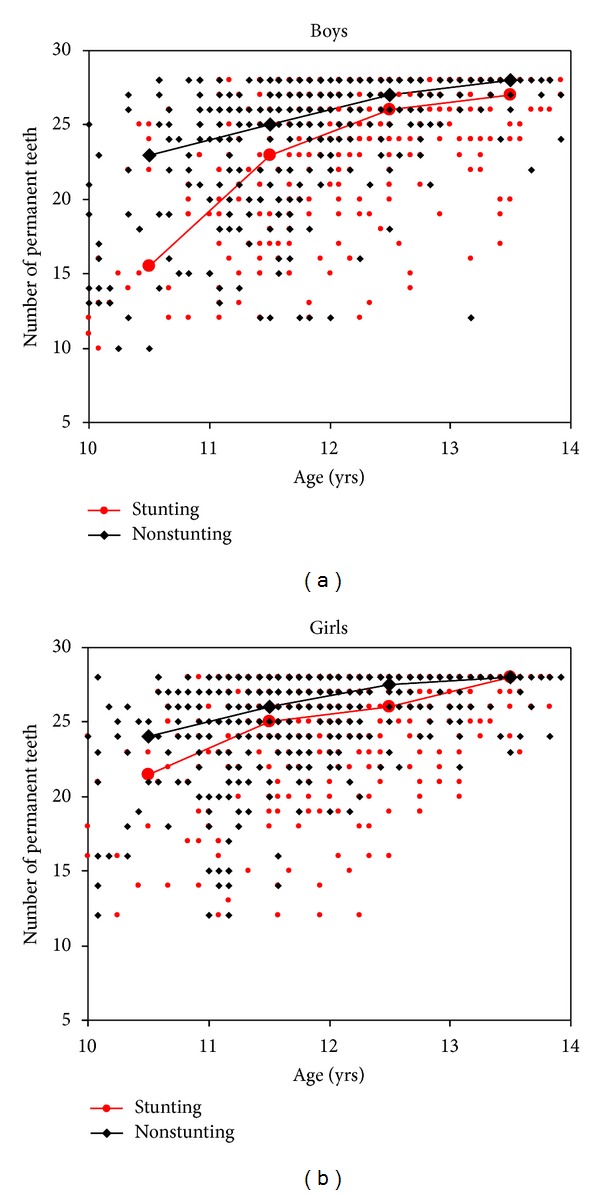
Relationship between number of permanent teeth and age in stunted and nonstunted (a) boys and (b) girls with interpolation lines between median values in age groups (the midpoint of the interval).

In both sexes the number of PT was significantly correlated to the HAZ as well as to the BMI-*z*-score. In boys, the correlation coefficient for the BMI-*z*-score (*r* = 0.146) was higher than that for HAZ (*r* = 0.098), and in girls, HAZ (*r* = 0.153) was closer correlated to the number of PT than the BMI-*z*-score (*r* = 0.106). Thin and stunted students had on average one to three fewer teeth than their nonthin and nonstunted contemporaries, respectively (Table 2). These differences were significant in the overall group of boys and girls and more obvious concerning stunting in the different age groups. In boys as well as in girls, the differences tended to be the result of a delay in tooth eruption in thin and stunted students since the differences decreased with increasing age (Figures 1 and 2). In 13-year-old girls, all PT (*n* = 28) were erupted regardless of their nutritional status, but thin and stunted boys did not have all PT yet at this age. They had on average one tooth less than normal boys. 

## 4. Discussion

The prevalence rates of malnutrition among the Filipino adolescents examined are high. Less than half of the students (45.1%) belonged neither to the thin nor to the stunted group, while 10.6% were both thin and stunted, and 44.3% were stunted or thin (35.5% stunted/nonthin; 8.8% thin/nonstunted). Boys were significantly more affected from malnutrition than girls. 

The joint UNICEF-WHO-World Bank prevalence estimates of child malnutrition show that stunting is widespread among children under five in developing countries [26]. In 2011, 165 million (26%) boys and girls (birth to 60 months) were stunted and 60% of the under 5-year-olds were underweight. The joint database shows that in South Asia prevalence rates of stunting and underweight are extremely high in comparison to other regions of the world [26].

The high prevalence rates found here for Filipino students are similar to that of the 7th National Nutrition Survey (NNS) in the Philippines in 2008 [27]. This survey, which belongs to a series of nationwide studies undertaken by the Food and Nutrition Research Institute (FNRI), revealed that in every 100 individuals (aged 11 to 29 years), 17 were underweight. Data for height-for-age demonstrated that 33.1% of the 6- to 10-year-old children were stunted. Our finding, that both underweight and stunting were more prevalent in boys than in girls, was confirmed by these nationwide surveys. Additionally, these surveys disclosed that the nutritional situation in the Philippines has not much improved from 2003/2005 levels. The underweight prevalence in adolescents increased from 16.0% in 2005 to 17.0% in 2008 [27]. 

Direct comparisons of prevalence rates of malnutrition found in our study and that reported from the NNS in the Philippines are difficult because in the latter malnutrition in 11- to 19-year old children and adolescents was assessed according to the CDC growth reference charts [27]. Such comparisons between different surveys, especially in children older than 5 years, are in general hardly possible because sample age, growth references, and cutoff values vary considerably between different surveys. Moreover, the WHO growth reference data for 5- to 19-year-olds were slightly modified after 2006 (WHO 2006), and the application of *z*-scores were recommended, but formerly percentile values were widely in use for classification of the nutritional status. This change alone may cause slightly lower rates of thinness because the BMI cutoff value of −2 SD to define thinness is equal to the 4.6th percentile. However, in many previous studies the 5th percentile was applied. In 2011, UNICEF and WHO initiated a process of harmonizing data and statistical methods to derive child malnutrition estimates for comparing children under five [26]. Such data harmonizing should also be done for children older than 5 years old and for adolescents, enabling meaningful comparisons at the global level.

The results of the present study indicate the association between the nutritional status and the eruption of PT defined by the number of teeth. We could demonstrate that thin and stunted Filipinos had on average one to three fewer teeth than their nonaffected peers. These findings support the results of a recent study on 11- to 13-year-old Haitians, where a delayed eruption of PT was associated with stunting [23]. While, few previous studies confirmed such relationship [8, 9, 28, 29], one study reported that the association between number of erupted PT and height and weight at a given age disappeared when Gambian children were classified regarding their weight and height [30]. In contrast, a consistent relationship between delayed primary tooth eruption and malnutrition was found in numerous studies performed in younger children [22, 31–35].

Alvarez et al. [33] stated that tooth eruption timing, being generally multifactorial, is strongly associated with the skeletal growth. Consequently, factors impairing skeletal growth as chronic diseases, infections, or insufficient energy, protein, or micronutrient intake should also alter the tooth eruption timing. In a short summary of the causal metabolic processes, which might explain the link between delay in tooth eruption and PEM, the importance of early childhood metabolic programming in this relationship was highlighted [23]. Psoter et al. [23] stated that intrauterine nutritional deprivation as well as malnutrition in the first year of life may cause a “metabolic shift” that affects later growth and development including oral development. This assumption was confirmed by studies which reported the relationship of factors such as short gestation, low birth weight, or postnatal weight gain to delayed tooth eruption [36–41]. Most of these studies suggested that risk factors for intrauterine and postnatal growth retardation like maternal undernutrition or systemic disarrangements may affect not only growth processes, but also the emergence of the primary and permanent dentition as well. 

Malnutrition can have an early onset during intrauterine life and, therefore, the high proportion of nutritionally at risk pregnant women (26.3%) and underweight 5-year-olds (26.2%) registered in the 7th NNS of the Philippines [27] may be related to the high rates of stunting in Filipino children. This early poor nutrition and/or repeated episodes of infectious or chronic diseases lead to low height-for-age or stunting which generally occurs during the first two years of life [42]. However, several studies suggest that stunting that persists later in life and affects the children is largely irreversible [43–45]. Stunted individuals show delayed motor development, and impaired cognitive function and do not reach their optimum size as adults [46], but they are not necessarily thin. In numerous (stunted) individuals, malnutrition can occur during lifetime and can predict continuing nutritional stress. Thinness (expressed as low BMI-for-age) arises from an inadequate intake of dietary energy. It reflects a combination of acute and chronic undernutrition, although it cannot distinguish between them. Both stunted and thin children are more likely to carry long-term health risks.

There is empirical evidence that early (prenatal) critical stages exist in which environmental factors affect tooth development and later tooth eruption. Tooth development begins usually between the sixth and eighth week of gestation. Both deciduous and PT enter the phase of mineralization before or soon after birth. Permanent canines and first premolars, which develop later, have their susceptible stage probably during the first post-natal years, while in the second premolars and molars, it occurs 2 or 3 years later [47]. Malnutrition in these critical periods may contribute to stunting and impaired dentition. Additionally, acute undernutrition may cause thinness and directly contribute to a delayed tooth eruption. 

Some remarks should be made with regard to the methodology of our study. Despite being suitable for investigating the association between stunting and thinness and the number of PT, the cross-sectional design used here is unsuitable for inferring causality. No information to early childhood malnutrition periods was available, and no conclusions concerning the causal links between malnutrition and delay of tooth eruption can be made here.

The findings presented here have methodological consequences on studies that compare data on dental caries experience between differently nourished populations during childhood and adolescence. Because the mean number of PT is lower and the retention period in the oral cavity is shorter in malnourished individuals at a certain age, caries experience (DMFT or DMFS scores) will be underestimated. Alvarez et al. [31] calculated a delay of the caries peak activity during the mixed dentition of 1.5 years in stunted versus nonstunted Peruvian children and of approximately 2 years in underweight versus not underweight subjects. The delay of PT should also lag the DMFS scores in malnourished children and is important to consider in age-related caries analyses and their global comparisons [23]. Particularly, in low-income developing countries, where both burden, malnutrition, and dental caries are highly prevalent in children, health professionals and policy makers should be aware of this relationship and should collaborate in preparing general health care policies [48]. Beyond that, delayed eruption of PT has several clinical consequences as tooth eruption time and sequences are important factors in dental treatment planning, particularly in those children who require interceptive, early orthodontic treatment as well as endodontic treatment in primary and PT. In addition, in forensic dentistry and in anthropology, delayed eruption of PT can confound the age estimation of children and should be considered in population with expected low nutritional status.

## 5. Conclusions

The observed delay of permanent tooth eruption in stunted and thin students should be considered if age-specific dental caries data from populations with different nutritional status are compared. This has especially practical significance for low-income countries with high malnutrition experience. Impaired physical growth and dental development have common risk factors that persist into adulthood. Regular monitoring of general health indicators such as height, weight, and dental development might help in targeting support programmes in developing countries. 

## Figures and Tables

**Table 1:** Characteristics of the 10- to 13-year-old Filipino students.

Variable	Boys (*n* = 711)	Girls (*n* = 843)	*P* value
Age (yrs)^a^	11.92 (1.08)	11.83 (1.00)	0.115^d^
10.00–10.99 yrs (*n*_all_/*n*_thinness_/*n*_stunting_)	85/25/26	83/15/30	
11.00–11.99 yrs (*n*_all_/*n*_thinness_/*n*_stunting_)	279/58/113	383/58/142	
12.00–12.99 yrs (*n*_all_/*n*_thinness_/*n*_stunting_)	245/58/142	277/41/139	
13.00–13.99 yrs (*n*_all_/*n*_thinness_/*n*_stunting_)	102/22/64	100/25/60	
Height (cm)^b^	135.5 (8.4)	137.4 (8.9)	<0.001^e^
Weight (kg)^a^	28.5 (6.0)	30.0 (9.0)	<0.001^d^
BMI (kg/m²)^a^	15.5 (2.1)	15.9 (2.5)	<0.001^d^
Number of permanent teeth (*n*)^a^	26 (5)	26 (4)	<0.001^d^
Stunting prevalence (%)^c^	48.5 (44.8–52.3)	44.0 (40.6–47.4)	0.082^f^
Thinness prevalence (%)^c^	22.9 (19.9–26.2)	16.5 (14.1–19.2)	0.002^f^

^a^Values shown are expressed as median (IQR).

^b^Values shown are expressed as mean (SD).

^c^Values shown are expressed as percentage (95% CI).

^d^Mann-Whitney *U* test—differences between sexes.

^e^ *t*-test—differences between sexes.

^f^χ^2^-test—differences between sexes.

**Table 2:** Number of permanent teeth among 10- to 13-year-old Filipino students according to thinness and stunting.

Number of permanent teeth^a^						
Age	Boys			Girls		
	Nonthinness (*n* = 548)	Thinness (*n* = 163)	*P* value^b^	Nonthinness (*n* = 704)	Thinness (*n* = 139)	*P* value^b^
10 yrs	22 (15.3; 26)	19 (14; 23)	0.056	24 (20.3; 26)	23 (18; 25)	0.402
11 yrs	25 (21; 27)	22 (18; 26.3)	0.032	26 (24; 28)	24 (21; 27.3)	0.009
12 yrs	26 (24; 28)	25.5 (24; 27)	0.206	27 (25; 28)	26 (24; 28)	0.223
13 yrs	28 (26; 28)	27 (24; 28)	0.202	28 (26; 28)	28 (26; 28)	0.882

total	26 (22; 27)	24 (21; 27)	0.002	26 (24; 28)	26 (23; 28)	0.024

	Nonstunting (*n* = 366)	Stunting (*n* = 345)	*P* value^c^	Nonstunting (*n* = 472)	Stunting (*n* = 371)	*P* value^c^

10 yrs	23 (16; 26)	15.5 (12.8; 22.3)	0.002	24 (21; 26)	21.5 (16.8; 25)	0.009
11 yrs	25 (21; 27)	23 (19; 26.5)	0.011	26 (24; 28)	25 (22; 27)	0.003
12 yrs	27 (25; 28)	26 (24; 27)	0.001	27.5 (26; 28)	26 (23; 28)	<0.001
13 yrs	28 (27; 28)	27 (24; 28)	0.015	28 (26; 28)	28 (26; 28)	0.814

total	26 (22; 27)	25 (21; 27)	0.010	27 (24; 28)	26 (23; 28)	<0.001

^a^Values shown are expressed as median (P25; P75).

^b^Mann-Whitney *U* test—differences between nonthinness and thinness.

^c^Mann-Whitney *U* test—differences between nonstunting and stunting.
